# Pre-verbal Children’s Participation in a New Key. How Intersubjectivity Can Contribute to Understanding and Implementation of Child Rights in Early Childhood

**DOI:** 10.3389/fpsyg.2021.668015

**Published:** 2021-08-06

**Authors:** Eystein Victor Våpenstad, Brynulf Bakkenget

**Affiliations:** ^1^Department of Psychology, Inland School of Business and Social Sciences, Inland Norway University of Applied Sciences, Lillehammer, Norway; ^2^Faculty of Social and Health Sciences, Inland Norway University of Applied Sciences, Lillehammer, Norway

**Keywords:** intersubjectivity, narrative, infant communication, infant participation, embodied meaning-making, psychoanalytic developmental theory, depth hermeneutic understanding

## Abstract

Children’s participation and involvement has increasingly been on the agenda for the last few decades. The right for children to participate was established in the United Nations Convention on the Rights of the Child (UNCRC). However, even though the UNCRC gives the right to participate to all children, national policy and practice seems to draw a line on verbal language and exclude pre-verbal infants from participation. The spur of this paper is to challenge the exclusion of infants, to describe how pre-linguistic children communicate their intentions, and to show how an understanding of children’s participation grounded in intersubjectivity, can inform and reframe the participation of all children as being fundamentally about close relationships with sensitive and containing adults who look within themselves for the voice of the child. The infant’s proto-conversational narrative communicates interests and feelings through sympathetic rhythms of what infant researchers have named “communicative musicality,” and it can surface in the mother’s narrative about the child and their relationship. Intersubjectivity oppose the monadic view of man as separate and left only to imitate others and claims that humans from the very start are intertwined in a fundamental thirdness of co-created reality. Infants are powerful communicators who actively engage in intersubjective relationships with their caretakers only days after birth, and newborns actively influence and even control the mental process of those who communicate with them. Early childhood participation then, would be to find within ourselves the voice of the child. A research project building on the theories and ideas described in the first part of the article, is presented.

## Introduction

Intersubjectivity is not exclusively confined to a declarative, metarepresentational third-person perspective. We are not alienated from the actions, emotions, and sensations of others because we own those same actions, emotions, and sensations (Ammaniti and Gallese, 2014, p. 9).

Children’s participation and involvement has increasingly been on the agenda for the last few decades. Children’s rights to participate are founded in the United Nations Convention on the Rights of the Child (CRC). However, even though the CRC gives the right to participate to all children, national policy and practice seems to draw a line on verbal language and exclude pre-verbal children from participation. In 2005 the CRC Committee made a thorough study of the ways in which infants and small children should be able to participate, and they strongly stated that infants have the rights to participate and be heard. The Committee on the rights of the child (2005) recommended to give the highest priority to the study and development of specific methods for infant participation. By “infant participation” we mean: having an influence on infants life-world, and on decisions that have bearing on infants both as individuals and as a (vulnerable) group in society. We think that infant participation should be an ongoing process and elaborate on this below.

Lyons-Ruth et al. (2017) describes the worldwide burden of infant mental health and paints a picture of utmost urgency for the world to take better care of infants and their human rights. Colwyn Trevarthen, leading infant researcher for over five decades, makes this warning explicit in his editorial for the European early childhood education research journal: “Any human community, large or small, primitive or civilized, that neglects to provide care and companionship to its infants and toddlers, responding to their affections, imagination and “zest” for both well-being and learning, will have cumulating problems with its health and behavior, and with its economy, too. Training intelligence and ability to work for an income is not enough. Even the youngest human beings have to be respected and accepted as whole reasonable persons, with all their impulses, feelings and habits, and preferred companions, as Comenius advised in the seventeenth century.” (Trevarthen, 2012, p. 310).

In the field of children’s participation, there is a promising tendency to include infants and to start to understand and figure out how even the youngest children should be included in the participation and citizenship paradigm (Ulvik, 2015; Horgan et al., 2017). In a recent publication, Hultgren and Johansson (2018), writing from a Swedish context, address the inherent rationalistic or positivistic view of participation “as an activity carried out by competent, responsible, reflexive individuals, that is, adult-like individuals.” (p. 2). Hultgren and Johansson challenge the views of “participation as something that concerns decision-making and that children’s participation is an issue of the degree to which they can share in decision-making.” (p. 1–2). The authors propose and describe a new model of participation based not on decision-making and verbal language, “but as an ongoing process” (p. 2) also including infants and pre-verbal children. This is a very good start, and the authors describe a nicely created program in a children’s library where young children participate and contribute to the agenda. The library staff actively set up a hermeneutical circle of reflection helping them to challenge their preconceptions of children’s participation and to be more sensitive and curious in their contact with the children. However, this study has one main deficiency: the fundamental relational and intersubjective basis of the human condition is not included. In a very recent publication, von Bonsdorff (2021) voices a similar critique of the modern discourse on infants and human rights. This deficiency, which we agree is typical for the recent literature on infant participation, is our point of departure in this article.

This article is an attempt to contribute from psychology, developmental and psychoanalytic, to the understanding of even small children’s right to participate and partake in common cultural exchange and meaning making. The understanding of participation should expand from a narrow legal and rationalistic endeavor, to a primarily relational and intersubjective process. As von Bonsdorff (2021) suggests, “we need a more holistic approach, which does justice to infants’ playful, interactive and affectionate initiatives.” (p. 37). We hope that our contribution can be to demonstrate how infant intersubjectivity can have a significant influence on understanding and practice of infant participation.

This article is twofold. Firstly we try to describe a theoretical foundation for infant participation. Secondly we describe a research project building on this theoretical foundation and having the goal of introducing a practical way of helping infants to participate concretely. We have the ambition that this article can contribute to an increased interest on how infants can have their rights to participate realized, in practices such as infant mental health, midwifery, child protection and public health services. But we also hope that our somewhat rhetoric art of stating explicitly the rights and possibilities of infant participation, can bring the needs of infants and small children higher up on the agenda of politics and decision making in society.

The authors field of work and research is infant mental health, child protection and public health. The first author is associate professor with a Ph.D. in clinical psychology and psychoanalysis, and is a clinical child psychologist and child psychoanalyst working with infants and their parents. The second author is a Ph.D. student, family therapist and clinical child and family consultant working with infants and parents in the context of midwifery and public health services.

Our epistemological point of departure is in line with modern psychoanalytic and psycho-social research emphasizing the subjectivity of the researcher, the continuous back and forth influence between research material and researcher, the relational or mutual creation of meaning and the importance of reflection on one’s own listening position (Reeder, 2002; Rustin, 2012, 2019; Hollway, 2015; Hamburger, 2017; Cummins and Williams, 2018; Salling Olesen and Leithäuser, 2018; Holmes, 2019). We try to do our research in line with psychoanalytic participant observation (Rustin, 2012, 2019) and mostly in opposition to a rationalistic, removed and (clinically) neutral registration and recording of behavior. In this context, our knowledge mainly comes from interaction and interpreting interaction.

### Infants Communicate Their Intentions–How Can Grownups Listen?

We want to describe how pre-linguistic children communicate their intentions, and to show how an understanding of children’s participation grounded in intersubjectivity, can inform and reframe the participation of all children as being fundamentally about close relationships with sensitive and containing adults who look within themselves for the voice of the child. We also want to give a preliminary description of a research project that could bring us closer to a specific method of understanding and tapping into the voice of pre-verbal children and thereby helping us to fulfill the human rights of even the youngest citizens.

Our argument rests upon an understanding of the human condition as basically two-person or intersubjective and therefore a main section of the article is devoted to a description of intersubjectivity and how this can help us to understand and develop a new model of infant participation.

Infant participation could not be about decision-making. We cannot ask a 6-month-old if she/he wants to live with mum or dad after a divorce. But we can be certain that the infant will be affected by the relationship breakup. Through interacting with the infant (or the infant together with his/her parents) and then producing a narrative about this interaction and the child, we can try to find the voice of the infant in our own words. The child will not reveal to us where he/she wants to live (mum or dad), but the infant will probably convey to us that he/she dislikes the present situation and his/her family being in ruins. The participation comes into action not as some sort of hybrid decision-making, but as an ongoing intersubjective process making grownups more sensitive to the child’s intentions and conscious of the infant’s perspective. This kind of broadened understanding of the situation can make the parents take into consideration that their infant also has something to say and that he/she will be affected by their decisions to a great extent. This is infant participation–that grownups learn to understand infants as communicative, that they must act as a companion in communication, and that they can find inside themselves the voice of the child. Grownups should get to understand that infants are not indifferent, but highly intentional and meaning making creatures, wanting to have their opinion heard and legitimized.

The discourse around children’s rights and participation, has usually been about what can be done or how can authorities intervene on behalf of children. But what really counts in child development and quality of life, is the relationship to primary caregivers. The primary relationship should be regarded as the most important arena for participation, influence and co-created meaning. We should not forget the continuous embodied interdependence of infancy. Infants use their whole body to communicate their intentions, what is called an “enkinaesthetic polyphony” (Stuart and Thibault, 2015). We cannot understand infants by investigating them from the outside using a rational view looking for static contents of their minds. We have to participate with the infant, engage in conversation and open up for the impact the infant can have on us. If we can create a culture where infants have the opportunity to engage us and we decide to meet them with openness, recognition and admiration, the number of co-constructing communicative events filled with meaning, will increase significantly.

Perhaps we should try to turn it all around, stating that participation is not so much about increasing the way children partake, but about grownups being much more involved with small children and infants, taking great care in the narratives we produce about the participation.

### An Intersubjectively Created Narrative

**FIGURE 1:**
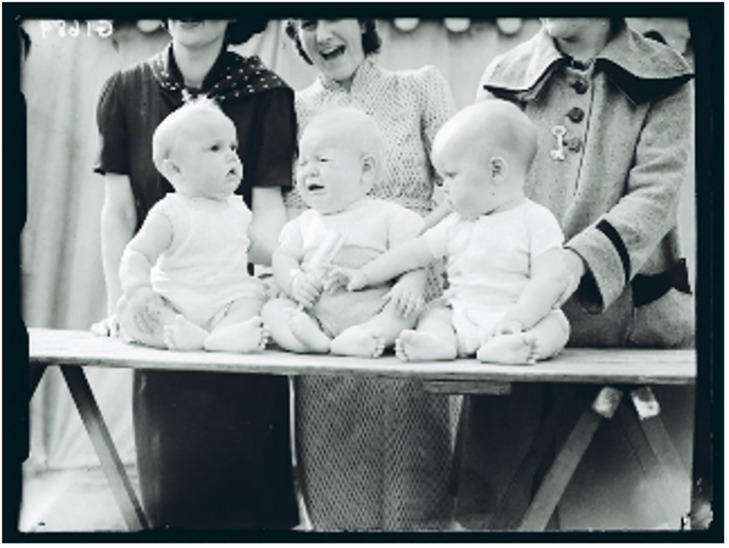
Reuben Saidman (1906–1967)/National Media Museum. Republished with permission from the National Media Museum.

These three human beings, put here on display (Figure 1), try in different ways to execute their rights as fellow citizens and members of society–we could use this as an example of citizenship and participation, at least a situation that gives us an opportunity to consider the rights of small children to participate and be heard.

Nobody likes to be put on display, infants are no exception. These three young fellows try in different ways to tell us their opinion about being part of a “cattle show.”

*The one to the left, gently patting his fellow citizen on the back and through identification with the distress of his neighbor shows the world with an annoyed look that this is not all right*.

*The person in the middle has reached the point of no return and is really pissed-off. There should be no doubt anymore–this member of society opposes the whole idea of being displayed and wants to go home with her mum. But mum is only laughing and there is no hope anymore of having her human rights accepted and respected*.

*The last child tries to help the crying baby to get rid of a troublesome stick or brush, and as you can see, supported significantly by her mum from behind, would like to throw the brush against the audience to move the center of attention elsewhere and to state clearly that the show has to end*.

This kind of spontaneously formulated narrative is of course heavily colored by the creator’s (first author) own subjectivity (one major part of this subjectivity is the ability and willingness to make the narrative). However, we think that this kind of narrative includes a core, containing some intersubjectively created meaning about the infants’ situation. These infants can have an impact on us provoking a narrative embodying their voices. The impact would of course be much more intense if we were present and had been interacting directly with the children, and would be even stronger if we had a caring function for one of them.

## Intersubjectivity

Like adults, infants behave as if they care about what’s going on in their world. Their close and caring relationships would be impossible without the intersubjectivity manifested by their prolific but directed emotional expressions. Infants are extremely engaging and intensely focused on making an impact. In the words of Colwyn Trevarthen (1979): “In any attempt to understand infants as communicators it must be noted that the effect of the emotional expression can only be interpersonal. Only another person capable of emotion can be influenced by an emotional sign.” (p. 323).

We will not go into detail on the huge amount of research that supports infant intersubjectivity but limit ourselves to a description of the phenomena and understanding of its implications. Research on infant intersubjectivity has been described in detail by (cf.) Trevarthen and Aitken (2001), Bråten and Trevarthen (2007), Zlatev et al. (2008), Bråten (2009), and Ammaniti and Gallese (2014).

It is tempting to use the seminal book *Before speech* edited by Bullowa (1979) as our starting point in grasping how intersubjectivity in developmental psychology can make a ground-breaking contribution to infant participation and citizenship. One of the very first, and widely cited, contributions by Colwyn Trevarthen, is part of this book. We have already quoted him above. Trevarthen’s chapter is called: *Communication and cooperation in early infancy: a description of primary intersubjectivity* and pointed toward the next four decades of research into the communicative abilities of even the youngest children.

Trevarthen (1979, 1993, 2001, 2011, 2015, 2016) has been one of the most influential and inventive researchers of infant development. His description of primary intersubjectivity has been a cornerstone of intersubjectivity theory and contemporary understanding of infant communication. Research on engagements between infants and parents proves that human beings are born with subjectivity and a drive for intersubjectivity. Trevarthen proposes that the infant is born with a receptive competence to grasp subjective states in other persons and to engage in conversation based on this competence.

The Norwegian expert on infant intersubjectivity, Stein Bråten, speaks of *altercentric-participation* (as opposed to egocentric). This is “the manifestation of an intersubjective capacity for participant perception, entailing that the perceiver resonates with what the other is doing or trying to do or say, as if the perceiver’s frame of reference were bodily centered in the other.” (Bråten, 2007, p. 117). Bråten and Trevarthen has been in close collaboration through the years, and together with others (cf. Beebe and Lachmann, 1988; Tronick, 1989; Kugiumutzakis, 1998; Meltzoff and Moore, 1998; Jaffe et al., 2001; Stern, 2004; Reddy, 2008; Beebe et al., 2010; Beebe, 2014) they have given us a widely recognized understanding of intersubjectivity and infant communication. Now is the time to use this knowledge in the interest of one of the most vulnerable groups in society, and to give them an increased opportunity to participate and have influence on their own condition (in accordance with the policy of the UNCRC).

The research referred to here has illustrated again and again that infant communication should not be regarded as more primitive and only a simple shadow of adult conversation because infants lack verbal language. Infants can express their wishes, needs and intentions in much the same way as we find among youngsters and even adults (Murray and Trevarthen, 1985; Bråten, 1998; Kugiumutzakis, 1998). In the words of Trevarthen and Aitken (2001, p. 6), summing up the research on infant intersubjectivity and communication: “Importantly, the behaviors selected to define the infant’s intersubjectivity–the ways the infant look, express their feelings in face and voice, how they gesture and move in rhythmic cycles to accept or reject contact–were homologous with behaviors that are essential to the elaborate intersubjectivity of all collaborative intentional activity in adult society, including live conversational language.”

### Infants Are Response-Able

The late Norwegian professor of psycholinguistics, Rommetveit (1998), said in response to the rationalistic and positivistic view of children’s participation and moral development: “The infant is …*response*-*able*, i.e., able to respond, but not (morally) *responsible* for its contributions to the interaction.” (p. 364; italics and underline in original). The spontaneous, not reflectively monitored, transcendence of the infant’s immature self into the feelings and intentions–and narratives–of the adult caretaker, is a pre-requisite not only for entrance into a community of meaning, but also for the development of moral agency.

Returning to the picture (Figure 1) of the display: One of the students of Colwyn Trevarthen, Bradley (2009), questioned the validity of information taken only from mothers or other adults with their infants, and went on to demonstrate the rich adaptations for communication, exploration of relationships and artful invention among infant peers from before 6 months of age. They (the infants) said: we are born “sociable,” not just for mothering. Infants communicate, they are interested in what’s going on in others, they have an impact on every sensitive human being, they are able to contribute and to respond. The company of an infant will be charged with intersubjectively created meaning. And if grownups take the task of putting into words what this interaction with the infant has done to them, the meaning can emerge and create a new potential for action and intention. This type of narrative can have an immense potential, as Trevarthen and Delafield-Butt (2013, p. 190) states: “In a narrative, separate psychological events or actions become one evolving experience, a product of an integrative action of the brain and body joining separate moments of conscious commitment and emotional evaluation in sequence to make a single and new project. That is the job of a narrative.”

### Intersubjectively Created Narratives

As Trevarthen and Delafield-Butt declare in the quote above: narratives are how we make our self-conscious agency meaningful to others, and this goes also for infants. The narrative influenced by the picture of the three young citizens, is an example of intersubjective communication. The narrative is a co-created story containing ingredients from the infant’s narratives as well. Delafield-Butt and Trevarthen (2015) states: “Narratives do not have to be linguistic. Understanding the pre-verbal origins of narrative is fundamental for understanding human cognition and culture” (p. 9). They also site Bruner (1990, p. 77): “Narrative structure is even inherent in the praxis of social interaction before it achieves linguistic expression.” The three children in the picture should absolutely be regarded as storytellers in the first degree. Their story ingrains my (first author) story.

Mental life, from cradle to grave, is an ongoing process of narration (Bruner, 1990). Our whole life is a continuous story-telling. We contribute heavily to our own life-story, except at the beginning and at the end. At the start and closure of life we are in the narrative mercy of someone else. There is a narrative already in place before we can start to develop our own, and this (pre-) narrative will influence significantly on our own agency and ability to continue the production of our story (MacIntyre, 1981; Ricoeur, 1992). When we enter the world, we are thrown (to use a term from Heidegger) into a world (or a narrative) where a community of persons already have started to tell the narrative of our life. Life narratives are produced in story-telling contexts. The story-telling context can produce a narrative that gives room for the infant’s own agency, or it can restrict the young child’s own influence. In the research project (described below), we will analyze the story-telling context of parents and professionals in conversation about the (coming) child, and try to find the child’s agency in the narratives produced there.

Another primary contributor to the famous book edited by Margaret Bullowa, was Bateson (1979). Her delicate observations and understandings of one mother-infant proto-conversation, illustrated that both mother and infant “were acting to sustain [the conversation] or to restore it when it faltered.” (p. 65). This mutual effort and responsibility to create and maintain the communication, reveals what Trevarthen and Delafield-Butt (2013) names “a fundamental *motive force* for intersubjective connection, for sharing what is in mind, and for making sense of existence in a human-made world.” (p. 191; italics in original).

The newborn expects the responsive company of others and this is even present before birth (Piontelli, 1992; Meltzoff and Prinz, 2002). Striking evidence of a readiness for communicating with others comes from detailed observations of close encounters with infants just hours old (Meltzoff and Moore, 1998; Trevarthen, 2011). In one illustrating example, a newborn was observed in dialogue with her father 4 weeks after her premature birth at 27 weeks’ gestation (Trevarthen, 1999). This case clearly demonstrates the inborn expectancy for human company and communication (Trevarthen and Delafield-Butt, 2013). Infants insist on communicating and wish for conversation with other human beings from the start of life.

Trevarthen and Aitken (2001) describes what they call a “shared narrative awareness” where the infant can also be the leading part. Together with a parent or another familiar person, the infant “can take the role of instructor or informer to the adult’s communications […] The infant gives an external curriculum of motive changes for the parents’ intuitions to follow, and this curriculum changes intrinsically as the infant develops. It is not simply a reflection of what the infant has been taught.” (p. 16). Infants are not compliant imitators of familiar adults who engage with them in joyful celebration, they are active and stimulating performers often making the initiative and leading the other (Schögler and Trevarthen, 2007).

When we are seeking a narrative understanding of the other’s feelings and intentions, we are not just characterizing the other’s inner life in the form of causally working mental states. What we try to understand is much more prolific and comprehensive. We try to grasp the other’s (infant or adult) reasons and causes as they reveal themselves against the whole history and myriads of potentials. The narrative form is the best way to capture this rich and complex communication. When we try to understand another’s reasons, we are not revealing their discrete mental states, but their perspectives and responses as whole situated persons. To produce a narrative about an infant would not primarily be to create a description of what is “going on inside the infant’s head.” Instead, the narrative would tell a meaningful story about what is going on in the world surrounding the infant, and this is a shared world, we are also living in the same world and in the child’s life from moment to moment. This is why the narrative comes to us as a primary example of intersubjectivity, because it contains a direct form of understanding of the other’s life and responses. The adult narrative will be ingrained (and even dictated) by the child’s performance. Attending to a narrative framework activates our subpersonal mechanisms for imitative and emotional responding, because it embodies our engagement and creative, say life-giving, potentials (Currie, 2007). This type of understanding intersubjectivity can be given the name “second-person approach” (Ammaniti and Gallese, 2014). It tangents neuroscience and the discovery of the mirror neuron. Vittorio Gallese had a central role in the research on mirror neurons and in several articles (Gallese, 2001, 2005) he proposes that our capacity to share experiences with others comes from the formation of a shared meaningful interpersonal space. Gallese names this shared capacity a “shared manifold,” a primitive, but, nevertheless, significant and fundamental we-centric space.

The grownup’s narrative about a shared life-world with an infant, should then not be understood as a “story about what’s inside the infant,” or what’s inside the grownup’s reverie or fantasy. We should take the narrative as a primary way of the child to put his/her reasons and intentions into adult language. We could say that the grownup speaks on behalf of the child, but without doing this consciously, more like a “medium” without the magic, this is serious business. Intersubjectivity holds that it is not possible, or even preferable, to sort out what’s mine and what’s yours, but that it is co-created, more like a third (Ogden, 1994; Benjamin, 2018) entity or fact. From this we can postulate that the voice of the child rests in the voice of the adult. This can obviously come to a cartesian mind as a challenging mental ordeal. But it is very gratifying and significant in the day-to-day practical work with infants and their parents. And for the majority of parents caring for their newborn, this is no surprise.

An understanding of the rights of the child to participate grounded in intersubjectivity and intersubjectively created narratives, makes the way for including infants and toddlers, and bringing them closer to the table when society decides about their needs and wellbeing. If we as grownups could act more like “*companions in meaning making*” (Trevarthen and Aitken, 2001), the rich and comprehensive messages from our youngest citizens, could guide us in developing the “culture of a cooperative society.” (Delafield-Butt and Trevarthen, 2015).

## A Psychoanalytic Contribution

We start this section about the psychoanalytic contribution by presenting another illustrative example: let us enter the Greek myth of Oedipus where the shepherd Phorbas rescues the infant boy and thereby makes Oedipus able to fulfill his destiny. Hearing about an infant, mutilated by his own parents and left in the forest to die, surely has an impact on all of us. This is probably one of the main reasons why the myth has survived, and through psychoanalysis, has become part of folk psychology.

The Norwegian word for participation or user-involvement is “*medvirkning*.” The word has two parts “*med*” and “*virkning*.” The first part translates into “*with*” and the last part “*virkning*” means literally “*impact*.” So, the word “*medvirkning*” equals “*with (an) impact*.” Impact is the key word here. We need to understand the impact, or the sharing and intersubjective, dual, non-monadic way we as adults understand infants through the influence and effect they have upon us. They emotionally affect us. We do not need a theory-of-mind, we already have an innate ability to grasp what the infant conveys through our sensitivity and understanding as part of a common culture and community, as the Oedipus myth confirms. Psychoanalysis has understood this human existential constant and has made good use of it through the concepts of countertransference (Money-Kyrle, 1956; Brenman Pick, 1985/1997) and projective identification (Bion, 1962).

Safier (2000) talks about the “impact of having the baby in the room.” The impact is not always a pleasant one. The British psychoanalyst and pediatrician Winnicott (1958) wrote in his illuminating paper “Hate in the countertransference,” how “the mother hates her baby from the word go.” (p. 201). Winnicott explained how a mother deals with this: “The most remarkable thing about a mother is her ability to be hurt so much by her baby and to hate so much without paying the child out, and her ability to wait for rewards that may or may not come at a later day.” (p. 202).

Very often, the impact, communication and understanding happens unconsciously. Even if the situation is obvious and the intersubjective communication is straightforward, the adult does not immediately understand. This is an inevitable part of everyday life. Different degrees of sensitivity due to psychic state, personality, other demands, contextual support, postpartum depression, etc. will of course influence on the parent’s ability to deal with the impact of the intersubjective communication (Gratier et al., 2015). In research and clinical work one should of course try to give caregivers an open and attentive ear, and take into consideration their potential difficulties. If we want to understand the life-world of an infant trying to communicate, trying to have an impact, we have to be open and sensitive to the everyday ordinary struggles of parents and infants. And we should do what we can to support caregivers in their strive to understand what their infant tries to convey. Psychoanalysis makes an important contribution to our understanding of infant intersubjectivity, not the least through highlighting the conflictual dynamics and the unconscious functioning of the relationship between parents and children that is often undervalued in infant research.

### Psychoanalytic Developmental Theory

In his book, *The Present Moment in Psychotherapy and Everyday Life*, psychoanalyst and infant researcher Stern (2004, p. 81) says: “The essential point is that when people move synchronously or in temporal coordination, they are participating in an aspect of the other’s experience. They are partially living from the other’s center.” Modell (1993, p. 120) making an obvious connection between intersubjective infant research and modern psychoanalysis, states that: “mother and infant unconsciously replicate within themselves the affective experience of the other.” Modern psychoanalysis, often named the “relational turn,” suggests that subjectivity is interpersonal from the beginning and reject the intrapsychic conception of the mind sustained by psychoanalysis for almost a century.

Lyons-Ruth (1999), representing relational psychoanalysis, states that “Recent psychoanalytic theory has moved increasingly toward a relational, intersubjective and social-constructivist stance” (p. 576), and she introduces the concept “two-person unconscious” as an alternative to the Freudian intrapsychic “dynamic unconscious.” The two-person unconscious includes implicit or procedural forms of knowing. “Procedural forms of knowing are not only infantile, but are intrinsic to human cognition at all ages and underlie many forms of skilled action, including intimate social interaction” (ibid, p. 579). Procedural forms of knowing includes the understanding of infant communication. “The re-transcription or translation of implicit relational knowing into symbolic knowing (for instance verbal language), is laborious, is never completely accomplished and is not how developmental change in implicit or procedural relational knowing is generally accomplished” (ibid, p. 579). The process of creating adequate intersubjective recognition in development requires close attention to the infant’s initiatives in interaction, because through these initiatives, the child communicates his or her goals, motives, intentions and their meaning structures. Without recognition of one person’s (the infant) initiative communications by another (the parent), no intersubjectivity or dyadic regulation is possible. Therefore, Lyons-Ruth as a practicing psychoanalyst knows very well the “active negotiation and repairing of miscues, misunderstandings and conflict of interest” (ibid, p. 584), that are inherent in every psychoanalytic relationship between patient and analyst, and in every parent-infant dyad.

“The concept of projective identification is an important bridge between psychoanalysis and the intersubjective approach.” (Ammaniti and Gallese, 2014, p. 35). Through projection of thoughts, beliefs and parts of the self together with the change that takes place in the person receiving the projection, the first person is, one could say, actualized in the latter, and usually without any awareness of what is going on before the receiver discovers it in his or her own behavior, thinking or narrative. This is extremely relevant in the parent-infant relationship. Ordinary devoted parents attributes heavily into the child (Seligman, 1999). In addition, the other way around, the infant is extremely dependent on the use of projective identification as a primary way of communicating his or her intolerable state to the parent, and to receive back a digested version made comprehensible by the parent’s reverie and containing abilities (Shuttleworth, 1989). Infant and child development rests upon the relational dynamics of the child’s ability to communicate its mental state, and the impact it makes on the caregiver. “Understanding would then be synonymous with receptivity, reverie and alpha-function,” (Norman, 2001, p. 94).

We should also highlight the contribution of psychoanalytic infant observation (Rustin, 1989, 2012, 2019; Urwin and Sternberg, 2012) to our knowledge of infant communication and development. Through an enormous number of naturalistic observations of infants and toddlers across their first years of life, psychoanalytic infant observation has given small children a strong voice and made us understand how they participate and have an impact on us. Building on the classic contributions of Bick (1964, 1968), psychoanalytic infant observation maintains that “emotions, qua emotions, have to be *felt* in some way, even if in a very mild identificatory way, to be faithfully recorded by an observer. It is precisely this element of intersubjective communication (more controversially, ‘unconscious to unconscious communication’) that is reflected in the first-person account, with its use of ‘subjective’ and evocative language where ‘every word is loaded with a penumbra of implication’ (Bick, 1964).” (Price and Cooper, 2012, p. 57, italics in original). To ask parents to produce a narrative about their infant should therefore be a self-evident way of finding the voice of the infant (as we will elaborate on below).

Another major point made by psychoanalysis and psychoanalytic infant observation, is the same as Van Manen (1990) declares of every form of phenomenological inquiry: “we know too much.” We come to the encounter with an infant with too much pre-understanding, common sense, scientific knowledge and the like, and we can become unable to grasp the intersubjectively created question: “what is this new member of humanity trying to tell us?” We need what psychoanalysts call “negative capability” (Bion, 1970), a concept Bion borrowed from the poet John Keats. It comes to us as a capability “of being in uncertainties, mysteries, doubts, without any irritable reaching after fact and reason.” (p. 125). And we should add the wise words of Symington and Symington (1996, p. 169) when they say that “Negative Capability is not an immediate mental discipline to be engaged in just prior to the session, but rather a way of life.” This is the way of life that psychoanalytic infant observation and infant intersubjectivity can teach us, and it is the way of life that can give our youngest citizens a stronger voice through our lenient openness for what they really have to say. Through our negative capability we can continue to be susceptible and open to the fact that every word in our narratives about infants is loaded with a penumbra of implication, as Esther Bick told us.

## A Method for Tapping the Voice of the Infant

In the tradition of psychoanalytic infant psychotherapy (Norman, 2001, 2004; Paul and Thomson-Salo, 2014; Safier, 2000; Salomonsson, 2014), “[t]he central therapeutic mechanism is thought to lie in trying to understand the infant’s experience from the infant’s point of view and conveying to parents and the infant that the infant has a mind of their own, with their own understanding of their experience.” (Paul and Thomson-Salo, 2014, p. 8). And we could add: trying to convey that the infant participates and want to be heard and to have an impact. The method is already there, developed from psychoanalytic infant observation and psychotherapy, and intersubjectivity gives us the theoretical, scientific and philosophical foundation to start tapping for the infant’s opinion. In this section, we will describe a research project designed to find the infant voice in narratives made by parents. The research is part of the second authors Ph.D. project at Inland Norway University of Applied Sciences and is done in cooperation with the public health services and midwifery section of Kristiansund municipality on the west coast of Norway. The project set off in early spring of 2021. The first author is supervising the project and takes part in the interpretation and understanding of the data material.

### Research Project: How Parents and Professionals Can Become “Companions in Meaning Making”

Trevarthen and Aitken (2001) in their scholarly paper on infant intersubjectivity, describes how a parent can become a *companion in meaning making* through primary (and later secondary) intersubjectivity. This companionship is a real and very effective form of informed action and engaged empathy, and our research project, has as its main goal to find and illustrate this very powerful way of intersubjectivity and infant participation.

The research project involves three distinctive stages. First, as part of a semi-structured interview with a professional (health visitor, midwife) before, and again around 6 months after birth, parents is encouraged to make a narrative about their child. The narrative can be oral or written. Both interviews will be videotaped and the audio will be transcribed. Between 15 and 20 parents will be included in the project, primarily mothers, but also the fathers narrative is of interest to us. Second, the parental narrative is interpreted according to a depth-hermeneutic method (Bereswill et al., 2010), called “scenic-narrative microanalysis (SNMA)” (Hamburger, 2017), based on the theories of scenic understanding first developed by the German psychoanalyst and sociologist Lorenzer (1970, 1986). Third, videotaped interplay between infant and parent at around 6 months after birth, is analyzed using the parental embodied mentalizing (PEM) instrument (see below).

Temporally coordinated expression (coordinated rhythm, prosody and interactive dynamics) forms the basis of a spontaneous communicative musicality in the first months of life (Malloch, 1999; Trevarthen, 1999; Gratier and Apter-Danon, 2009). Musically lived-through narratives can inspire and (at least partly) be reproduced after the interaction. The meaning making comes through the passing of intentions and emotions between infant and parent in “proto-narrative envelopes” of vitality (Stern, 2010). When a parent (or professional) who has been engaged in conversation and/or companion with an infant, elaborates in verbal story-making or singing, the grownup person makes sense in their own language, of the intentions and prospects delivered from the child through the proto-narrative envelopes, as “a semiotic experience for consensual understanding” (Delafield-Butt and Trevarthen, 2015, p. 12). In our project, on all three stages, we would especially look for the rhythm, pulse or prosody that makes an impact on us.

### The Rationale for Asking Parents to Make a Narrative Both Before and Again After Birth

Martiìnez Quintero and De jaegher (2020) speaks about pregnancy as a type of intersubjective relation between mother and fetus. Both mother and fetus participate in a kind of minimalistic sense-making, trying to figure out how to grow and develop in the new environment, seeking a kind of agency to exist and maneuver, for the sake of a common survival. It‘s a kind of embodied sense-making, forwarding some interesting questions about fetus consciousness and/or intentionality (Hata et al., 2015). Even though this may be open questions, we follow the line of Delafield-Butt and Gangopadhyay (2013) when they say that the fetus shows an embodied kind of primary intentionality that develops from early in pregnancy. We consider this to be a kind of “not one, not two” but a cooperative system “that emerges as an autonomous relational organization” (Martiìnez Quintero and De Jaegher, 2020, p. 14).

The coming mother’s experience of pregnancy and the growing fetus inside her, is of great interest in our project. Do the mother experience herself mainly as a container for the fetus or does she consider the fetus to be a part of her maternal body? (Kingma, 2019). The first may lead to a feeling of alienation, the latter may lead to an experience of cooperation between mother and fetus. We would like to call this cooperation “the birth of intersubjectivity,” and it happens before the physical birth. We assume that the mother’s experience of intersubjective partnership in sense-making with the fetus, can influence significantly on the quality of narratives about the infant after birth. We presume that the narrative made by the mother-to-be can reveal traces of this dichotomy: container or partnership (of course, in real life it will not be dichotomous, but more like a dynamic continuum). And we presume that the mother’s experience of cooperation with the fetus will affect the cooperation of sense-making with her infant after birth.

### Scenic-Narrative Microanalysis

“Scenic-narrative microanalysis assumes as a principle that meaning itself, and not only in the field of human psychology, is a relational phenomenon.” (Hamburger, 2017, p. 168). The meaning we are looking for is both created (in the parent-infant dyad) and understood (in the research group struggling to find the meaning-making narrative) relationally. SNMA was first developed, as a central part of the Yale video testimony study (Laub and Hamburger, 2017; Hamburger, 2017), to analyze and interpret videotaped interviews of Holocaust survivors. In our research project the data material to be analyzed, comes in the form of audio or videotaped interviews containing parental narratives.

The depth-hermeneutic method of interpretation consist of an intense group process with challenging negotiations to identify scenic or intersubjective moments-of-meeting (Stern, 2004) in the narrative material. The narrative material will be transcribed and made ready for the research group to dive thoroughly into, not only looking for the manifest verbal accounts, but also searching for hidden treasures in the narrative expression. Our presumption is that we can find some traces or even a clear resonance of the infant’s voice inside the parents’ narrative, and that we can find a qualitative development in the narratives ability to carry the infant’s voice from the time of pregnancy to the time of interaction a few months after birth.

Based on Hamburger (2017), the interpretive process of SNMA in our project, takes place on four consecutive steps or levels:

(1) Every single researcher (or member of the research team) expose her/himself to the transcribed raw material including the parental narrative. The researcher underlines and documents her/his transference reactions to the material.
(2) In one or two meetings of the research group, the reactions or provocations (see below) of each individual member, is discussed and compared. Most important: the group reactions and “moments of heated debate” are also recorded by the main researcher taking the minutes.
(3) In a new meeting of the research group, the minutes from the first meeting is presented and debated, again to identify and negotiate provocations (see below).
(4) The main researcher then makes a conclusive discussion of the material from the group meetings.

In our research project the main outcome produced, and which we assume would contain traces of the infant voice, is the minutes from the research group meetings and the conclusive discussion of the material.

### To Identify and Negotiate Provocations–the Backbone of Scenic-Narrative Microanalysis

The narrative approach is especially well suited to study and look for intersubjectively created signs of communication. Parental narratives infect the research group and elaborates in the collective continuous process of negotiation and interpretation. At one point we tap into this ongoing and neverending process to look for the voice of the infant.

According to Lorenzer (1970, 1986), and emphasized by Bereswill et al. (2010), Leithäuser (2013), and Hamburger (2017), the interpretation and understanding of a text should be based on the identification and following of “provocations.” The text could be any type of qualitative research material, including interviews and narratives (Bereswill et al., 2010; Hamburger, 2017). The researchers in our project are looking for the surprising, worrying, disturbing, confusing, irritating (and so on), parts of the parental narrative. And in the group meetings of three or four researchers, their respective provocations are discussed and debated. This debate can sometimes become quite heated, and this illuminates and reveals, through the researchers’ transferential potential, important intersubjective aspects of the parental narrative. This method is similar to the familiar practice of psychoanalytic infant observation where the main point is to observe and reflect on one’s affective (embodied) responses to presented material in the infant observation seminar (Rustin, 1989, 2019; Hollway and Froggett, 2012; Urwin and Sternberg, 2012; Music, 2017). Swedish psychoanalyst and infant researcher, Salomonsson (2014) names this interpretative work “*adultomorphizing*.” He states: “I therefore suggest we understand babies via a *qualified adultomorphizing*, namely, by reclining on *analogic representations linked to our own bodily experiences*.” (p. 38; italics in original). And he continues: “Once, when they were created in our infancy, they copied our affects’ gestures and contents. Today, in front of the baby [or the narrative about the baby], we recognize the similarity between his behavior and our representations.” (p. 38). The basic assumption is that the identification, understanding and truth of the provocation, does not only reside in the single receiver. Provocations are collective “in the sense of shared socio-cultural meanings drawing on the necessarily social quality of collective experience embedded in interaction forms.” (Hollway, 2015, p. 131). This gives us the foundation to claim that the infant voice can be found in the parental narratives, if we are willing to endure the demanding process of finding and negotiating what provokes us there. Or we could say: The infant has an *impact(a)* on the parent, the parent produces a narrative in a story-telling context together with the midwife or health-worker, and this narrative has an *impact(b)* on the researcher(s). Our presumption is that *impact(a)* and *impact(b)* will be related and this connection makes it possible to find the voice of the infant in the provocations that turns up in the research group meetings.

### Parental Embodied Mentalizing

In our upcoming research project, we will also use an instrument developed by Shai et al. (2017), Shai and Meins (2018) called PEM. PEM stands for “Parental Embodied Mentalizing” and is both the name of the instrument and the name of an important developmental and relational phenomena in the early interaction between infants and parents, which the instrument measures. Shai and Belsky (2011, p. 187) states that: “PEM is defined as the ‘parental capacity to (a) implicitly conceive, comprehend, and extrapolate the infant’s mental states (such as wishes, desires, or preferences) from the infant’s whole-body kinesthetic expressions and (b) adjust one’s own kinesthetic patterns accordingly”’. The main contribution of PEM, as a clinical and research instrument, is to uncover and operationalize parental reflective function (PRF), but moves beyond parents’ verbal and declarative capacities, toward an embodied “enkinaesthetic polyphony” (Stuart and Thibault, 2015). In accordance with the scientific foundation of our research project (as outlined in this article), PEM builds on the notion that “the development of children’s sense of ownership and agency at the embodied level necessitates the interpersonal encounter, mediated by PEM.” (Shai and Belsky, 2011, p. 187). Co-created narrative engagements between infant and parent, forms intersubjective events rooted in the sensorimotor (and thereby mostly unconscious) domain. PEM can help us trace these intersubjective events in a controlled and standardized way. The PEM analysis is based on the interpretation of a videotaped 10-min play sequence between infant and parent. The second author will do the filming. The play sequence is interpreted according to various embodied categories: pacing, direction of the body, tempo, space, pathway of movements, tension flow, initiative and so on. PEM usually generates an overall rating of parent sensibility. But in our research project, the different categories of embodied interaction highlighted by PEM, will be emphasized. We want to look for clues or traces in the embodied cooperation between parent and infant, that adds something to the depth-hermeneutic analysis (SNMA) of the narratives. Both PEM and the semi-structured conversations between parents and midwife/health visitor, are cooperative sense-making situations, and can therefore contribute and create narrative data suitable for the qualitative search for the voice of the infant.

The PEM classification rests upon the idea that the parent-infant interaction is an ongoing common meaning-making (a non-verbal narrative) and is comparable to the concept of communicative musicality (Malloch, 1999).

In using PEM, the professional or researcher analyses the child-parent interaction from an “outside” position, meaning that the researcher should not take into consideration the emotional (or personal) reaction in his/her own mind. PEM can be seen as a type of “exterior” analysis; looking on the outside to make a judgment. On the opposite, the depth-hermeneutic method; the SNMA, puts the emphasis on the emotional (or personal) response of the researcher and can therefore be regarded as a type of “interior” analysis; looking inside the researcher’s mind to make a judgment.

To compare a PEM score with a depth-hermeneutic analysis of parental narratives, can be challenging because they come from quite different epistemologies. Even so, we want to use a combination of the two sorts of empirical data in a mixed methods design, because we think that the exterior and the interior is not necessarily mutually exclusive. They may be “two sides of the same coin,” and at least that is what we want to investigate.

In our research project we want to compare the PEM analysis of each individual parent with their narrative. We are very much in favor of the PEM paradigm and instrument, but would like to develop a way of highlighting the infant’s contribution and involvement through a method, or more precisely, an eye-opener, that can easily be put into use. The narrative method, as described above, is all about telling stories and diving hermeneutically into those stories about the infant and the interaction. For instance, it can be made as a specific layout, where a professional interacting with an infant, directly afterward compose a narrative about the infant and the interaction, and with the help of a colleague or two, interprets the narrative, looking for the infant’s voice in the “provocations” emerging between them.

## Conclusion

Let us end with a quote from Delafield-Butt and Trevarthen (2015, p. 13), ending their article in *Frontiers in Psychology* like this: “Thus life stories with their intrinsic narrative vitality create a store of experience, memories, understanding and purpose–the culture of a cooperative society.” One major cooperative society–the United Nations, has a Convention for the rights of the child. This convention asks for a definite way of involving infants, the youngest citizens, and to let them use their narrative vitality to create in our culture a store of experience, memories, understanding and purpose. The only thing we as grownups have to do, is to lend them our minds and bodies, to use in the symbolization and transformation of their narratives into verbal language and constructive action on their behalf. If we understand, respect and believe in the foundational structures of intersubjective communication, we should take very seriously telling stories or singing songs about our infant members of society. Their participation, their voice, can surface in our verbal or musical narratives, and we should help each other to find them there.

## Data Availability Statement

The original contributions presented in the study are included in the article/supplementary material, further inquiries can be directed to the corresponding author/s.

## Author Contributions

Both authors listed have made a substantial, direct and intellectual contribution to the work, and approved it for publication.

## Conflict of Interest

The authors declare that the research was conducted in the absence of any commercial or financial relationships that could be construed as a potential conflict of interest.

## Publisher’s Note

All claims expressed in this article are solely those of the authors and do not necessarily represent those of their affiliated organizations, or those of the publisher, the editors and the reviewers. Any product that may be evaluated in this article, or claim that may be made by its manufacturer, is not guaranteed or endorsed by the publisher.
